# Electroconvulsive therapy as life-saving in an acute catatonic syndrome associated with bipolar disorder: A case report

**DOI:** 10.1192/j.eurpsy.2021.2072

**Published:** 2021-08-13

**Authors:** R. Almeida Leite, A. Costa, J. Borges, J. Alcafache, A. Mesquita

**Affiliations:** Psychiatry And Mental Health, Baixo Vouga Hospital Centre, Aveiro, Portugal

**Keywords:** Catatonia, Electroconvulsive therapy, bipolar disorder, neuroleptic malignant syndrome

## Abstract

**Introduction:**

Catatonia is a neuropsychiatric syndrome characterized by an onset of a dysfunction in psychomotor activity and/or muscle tone, which may be associated with changes in consciousness, affect, and thinking. It is characterized by negativism, wax flexibility, catalepsy, mutism echolalia, ecopraxia, or stupor. It was first described in 1874 by Kahlbaum, who characterized it as specific motor disorder associated with different psychiatric disorders. Kraepelin and Bleuler restricted catatonia to a specific subtype of schizophrenia. However, the association between catatonia and other disorders, notably mood disorders, has been reinstated, including Bipolar Disorder. Its etiology is multiple and there are two severe forms: Neuroleptic Malignant Syndrome (NMS) and Malignant Catatonia (MC). These are syndromes that present high mortality, and the health professional should be aware of its etiology, signs, symptoms, evaluation and treatment.

**Objectives:**

The aim of this work is to present a clinical case of MC, who was sustained by literature included on scientific platforms.

**Methods:**

Case report

**Results:**

It is essential to recognize the different clinical presentations of catatonia, taking into account that these are psychiatric alterations in which urgent intervention is justified. In the presented case, the use of antipsychotic medication has worsened the motor function and its suspension, associated with the introduction of lorazepam, resulted in a slight improvement. The electroconvulsive therapy was the last resort, fully succeeded.

**Conclusions:**

The relationship between SMN and Catatonia/MC remains nuclear from a psychopathological and pathophysiological point of view. Nevertheless, there is general agreement that catatonia represents a very significant risk factor for NMS.

**Disclosure:**

No significant relationships.

